# Explainable AI for unveiling deep learning pollen classification model based on fusion of scattered light patterns and fluorescence spectroscopy

**DOI:** 10.1038/s41598-023-30064-6

**Published:** 2023-02-24

**Authors:** Sanja Brdar, Marko Panić, Predrag Matavulj, Mira Stanković, Dragana Bartolić, Branko Šikoparija

**Affiliations:** 1 BioSense Institute - Research Institute for Information Technologies in Biosystems, University of Novi Sad, Novi Sad, Serbia; 2 Institute for Multidisciplinary Research, University of Belgrade, Belgrade, Serbia

**Keywords:** Computer science, Machine learning, Pollen

## Abstract

Pollen monitoring have become data-intensive in recent years as real-time detectors are deployed to classify airborne pollen grains. Machine learning models with a focus on deep learning, have an essential role in the pollen classification task. Within this study we developed an explainable framework to unveil a deep learning model for pollen classification. Model works on data coming from single particle detector (Rapid-E) that records for each particle optical fingerprint with scattered light and laser induced fluorescence. Morphological properties of a particle are sensed with the light scattering process, while chemical properties are encoded with fluorescence spectrum and fluorescence lifetime induced by high-resolution laser. By utilizing these three data modalities, scattering, spectrum, and lifetime, deep learning-based models with millions of parameters are learned to distinguish different pollen classes, but a proper understanding of such a black-box model decisions demands additional methods to employ. Our study provides the first results of applied explainable artificial intelligence (xAI) methodology on the pollen classification model. Extracted knowledge on the important features that attribute to the predicting particular pollen classes is further examined from the perspective of domain knowledge and compared to available reference data on pollen sizes, shape, and laboratory spectrofluorometer measurements.

## Introduction

In Europe as much as 40 percentage of population is affected by pollen allergy ^1^. The substantial costs from the disease itself or from productivity loss due to poor management of the disease exceeds several tens of billions euros per year ^2^. The burden of allergic disease can be limited by avoiding allergen exposure or timely therapy, which makes airborne pollen data and forecasts of utmost value both for patients and medical workers. Detection and quantification of airborne pollen have mainly been carried using standard volumetric method (EN16868) ^3^ which relies on labour intensive and lengthy manual identification of each bioaerosol particle under microscope resulting in at least 36 h delays for data availability. The stakeholders showed the need for the near real-time data ^4^ since it is expected to help patients relate better their symptoms to exposure thus providing a tool for more accurate timely diagnosis and for better assessment of therapy efficiency. In addition, like in meteorology, near real-time observations can be integrated into numerical models to provide improved spatial forecasts.

Recent technological developments proved that sampling and characterizing single bioaerosol particles is possible ^5,6^, however the discrimination is still challenging especially when pollen identification relies on complex signals representing both morphology and chemical composition of detected particles. The first attempt to resolve pollen classes from optical pollen monitoring based on time-resolved scattering and fluorescence was performed with artificial neural network and support vector machines classifiers ^7^. This classical machine learning approach demanded for extensive feature engineering steps for extracting properties of the measured signals. Further development of pollen classification models from chemical signatures and scattering information was accomplished with deep learning approach based on convolutional neural network (CNN) architecture ^8^.

Focus of our study is on data derived from the PLAIR Rapid-E instrument ^9^. The number of performed research and experiments is growing with the number installed devices across Europe ^10^ and beyond. Operational system in Serbia and Croatia ^11^ runs classification model of 26 aerosol classes. The transferability of the models is evaluated between models trained on data form device in Serbia and Italy ^12^. Database with over 100 thousand samples measured in Romania ^13^ is available. Case study in Lithuania utilized device for plant diversity investigation ^14^, while in Switzerland comprehensive comparison with other pollen monitoring approaches ^4^ was performed. Despite loads of data generated and extensive usage of classification models, mainly black-box models, there are no efforts directed toward explainability of such models.

Explainability of the models is essential for understanding and enabling further trust in Artificial Intelligence (AI)-based solutions ^15–17^. Challenges to explain AI models and to provide more transparent and understandable results become more complex with the fast development of AI itself. While simpler machine learning methods can be intrinsically interpretable and by design offer explanations of the decisions (i.e. decision trees ^18^) and other classical machine learning algorithms have been extensively explored from the aspects of the model interpretation ^19–22^, deep learning with millions of parameters distributed across deep layers in the model makes explanation harder to extract.

In this article, we present an explainable machine learning framework for unveiling the learned model for pollen classification. It is based on Integrated Gradients (IG), a gradient-based feature attribution method ^23,24^ that attributes the prediction of deep networks to their inputs. It provides instance level interpretations that we further aggregate to obtain overall model insights. Instance level interpretation highlights the classification-relevant parts of the input data, while overall level summarizes information from all samples to rank input features with respect to the predicted class. Developed framework can further help with answering questions why one model is better than another, what are the differences in the models learned on different devices, what are the novel insights concerning scattered light and fluorescence spectroscopy patterns of different pollen classes.

## Related work

Several automatic instruments for pollen classification emerged on the market ^25^ that are based on digital images or electrical signals from various types of sensors. Such instruments further demand machine learning algorithms to automate the process of pollen classification. Heterogeneous data recorded by instruments can be grouped into: (1) digital microscopy, (2) elastic light scattering, (3) light-induced fluorescence and (4) holography. By now deep learning was applied on all of these diverse data modalities and achieved good performance in terms of classification accuracy for many pollen taxa ^26^. Extensive comparison of methods spanning from classical machine learning algorithms to deep learning in the classification of microscopic images ^27^ demonstrated that deep learning methods are favored and produce better quality results. That is especially noticeable on larger data sets with a higher number of pollen taxa. Deep learning methods have been also used successfully to classify pollen types from holographic images of flowing particle ^28^ in a mobile and cost-effective sensor, as well as to classify pollen types from scattering images ^29^. Recent approaches in the field of automatic pollen classification utilize multi-modal identification, for example combining light-induced fluorescence and elastic light scattering data as in Rapid-E (Plair SA, Geneva, Switzerland) ^8^, or adding also holography on these as in Poleno (Swisens AG, Horw, Switzerland) ^30^. Such increased device complexity requires further advances in the machine learning models.

More complex deep learning architectures increased the accuracy ^31,32^, while combination of CNN autoencoders and self-supervised learning with small amounts of laboratory data were also explored ^33^. An interesting approach of using clustering algorithms to group pollen samples based on feature vectors resulting from neural network preprocessing ^34^ unveiled that fluorescent data modality played a more important role than scattering for separating fluorescent particles, but also confirmed that particle shape and size properties align with discovered scattering clusters. Apart from this indirect approach to extract knowledge learned by deep learning and one example of activation map obtained with gradient-based localization ^35^ in model learned on scattering images generated in the laboratory ^29^ there are no other explainable examples in pollen classification tasks.

Explainable AI (xAI) solutions for biomedical domain are attracting increasing scientific interest ^36^. Emerging applications include drug discoveries ^37^, cancer diagnosis ^38^, microbiome studies ^39^ and clinical decision support systems in pandemics ^40^. We believe that research community working on automatic pollen classification would highly benefit as well from xAI solutions providing insights into how classifiers make decisions. Although pollen classification models are yet to be unveiled by xAI, applications build upon principles of chemistry, physics or spectroscopy can demonstrate potential benefits of this methodology in the broader context. For example xAI for optical emission spectroscopy ^41^ in plasma-based processes unveil why model made certain predictions thus allowing to characterize the plasma and the spectra. Study of Gomez-Fernandez et al. ^42^ examined whether domain-specific characteristics are being identified by deep learning models on gamma spectroscopy tasks. In particular for the task of isotope classification evaluating the rationale behind the classification and testing if it is correlated to the isotope’s characteristic features is paramount in this highly regulated industry. In several chemical engineering applications xAI framework for mechanistic explanation generation ^43^ provided causal explanations by combining techniques from machine learning and symbolic AI techniques representing knowledge base. Furthermore xAI can be integrated into interactive visualization techniques to highlight regions of a molecule in complex notation of a chemical structure in order to reveal their influence over a predicted property ^44^. Another example is on ultrasound imagery where xAI approach helped in determining where to look for artifacts patterns ^45^. Discovered patterns were not previously known in the ultrasound literature.

Although our study focuses to demonstrate xAI principles on data coming from RAPID-E device other pollen monitoring instruments relying on different data modalities can unveil predictive models in similar manner and thus help discover the underlying principles.

## Data sources

**Figure 1:**
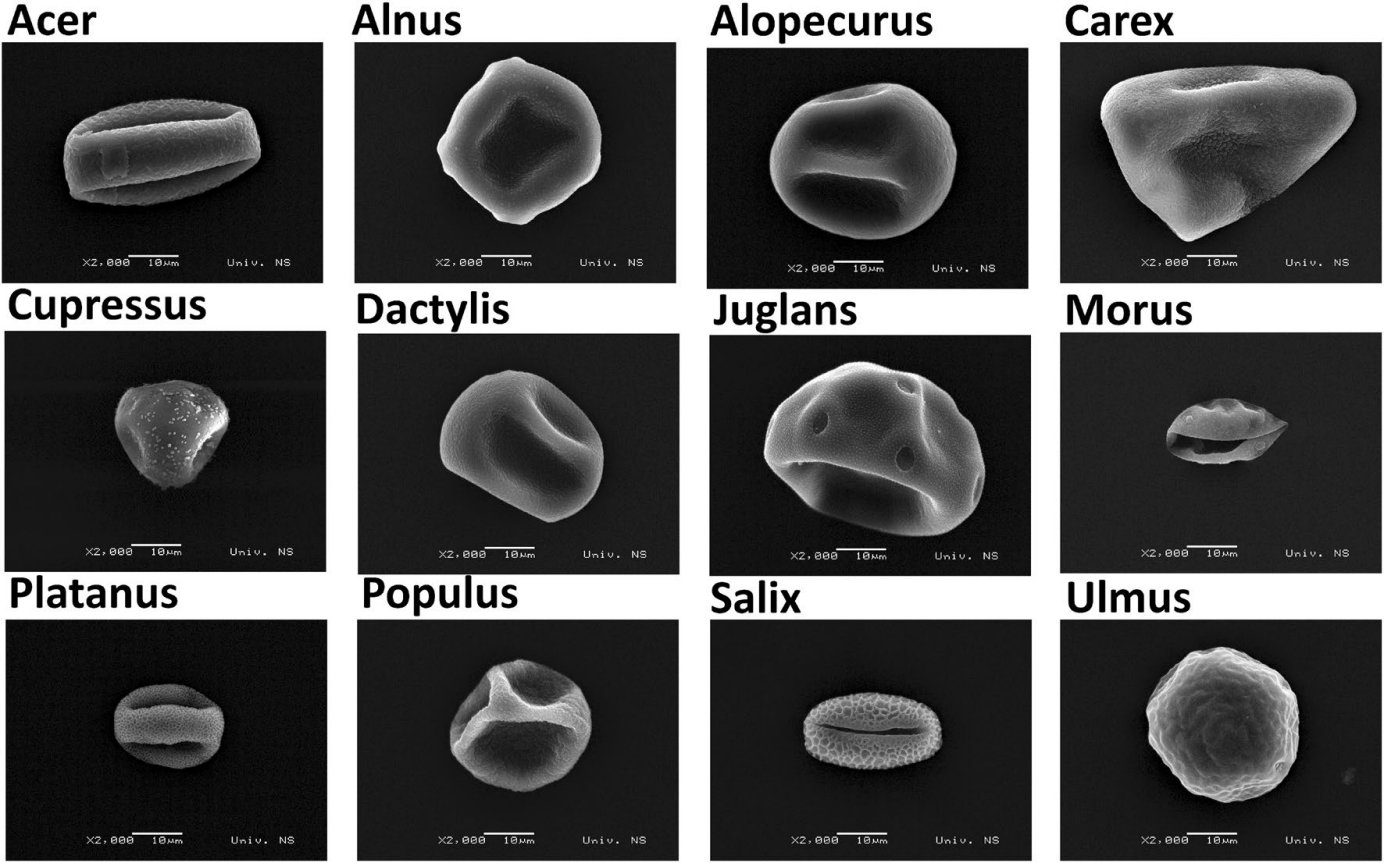
SEM micrographs in µm resolution of 12 examined pollen classes unveiling their diversity in shape and sizes.

Our study encompasses data that characterize airborne pollen. Selected 12 classes: (1) Acer, (2) Alnus, (3) Alopecurus, (4) Carex, (5) Cupressus, (6) Dactylis, (7) Juglans, (8) Morus, (9) Platanus, (10) Populus, (11) Salix, (12) Ulmus (Fig. 1) enabled us to concurrently examine collected pollen on different devices and link information extracted from different sources. High diversity of shapes and sizes as illustrated by scanning electron microscopy (SEM) micrographs enabled throughout analysis of data characterizing pollen classes coming from different sources. The pollen characterization data were measured using three different devices: Olympus BX51 bright field upright microscope at x400 magnification, HORIBA Fluororlog-3 spectrofluorometer and PLAIR SA Rapid-E single airborne particle analyzer. Microscope and spectrofluorometer were exploited to create reference data and extract distinctive features of examined pollen classes.

On the other hand, Rapid-E records three types of measurements representing morphological and chemical properties of the particles. The device is packed in a 40 cm $\times$ 34 cm $\times$ 73 cm box and weights about 20kg. The dimensions of the detection chamber including the length of the optical path of light scattering from the particles are not revealed by the manufacturer. When a particle enters the device, firstly a deep-UV laser interacts with it. The scattered light is collected with 24 detectors from −  45 to $135^\circ$ relative to the direction of the laser beam. If we take a look perpendicular to the laser this transforms to the −  45 and $45^\circ$ that we use to denote features. Angles range is further reduced in preprocessing to −  37.5 to $37.5^\circ$. The number of laser interactions with one particle depends on the shape and size of the particle, properties that directly influence its moving through the Rapid-E measurement chamber. The collected scattering signals are expressed as images of 24 $\times N$ pixels, i.e. 24 different angles and *N* number of interactions. After that, a second, deep-UV laser (337 nm) interacts with a particle. The fluorescence spectrum is recorded with 32 detectors representing a spectral range of 350–800 nm repeated eight times with an interval of 500 ns from the moment of excitation of the particle by the laser. Additionally, fluorescence lifetime is measured at four spectral ranges: 350–400 nm, 420–460 nm, 511–572 nm, and 672–800 nm for 48 ns with two ns temporal resolution.

Knowledge extracted from reference data was placed into the context of unveiled decision making process made by xAI from the model learned on Rapid-E data. SEM images served us to visually represent diversity of the examined pollen types, while on visible light microscope images pollen grains sizes were measured. With spectrofluorometer detailed spectral characteristics were measured. These data were not used to train, validate or test model learned on Rapid-E data, but to examine to what extent reference knowledge is being reflected in the unveiled deep learning model. This allowed us to question whether model indeed uses distinctive spectral and morphological properties extracted from reference data or builds its decisions on other features available in Rapid-E data.

## Results

### Model and classification results

**Figure 2:**
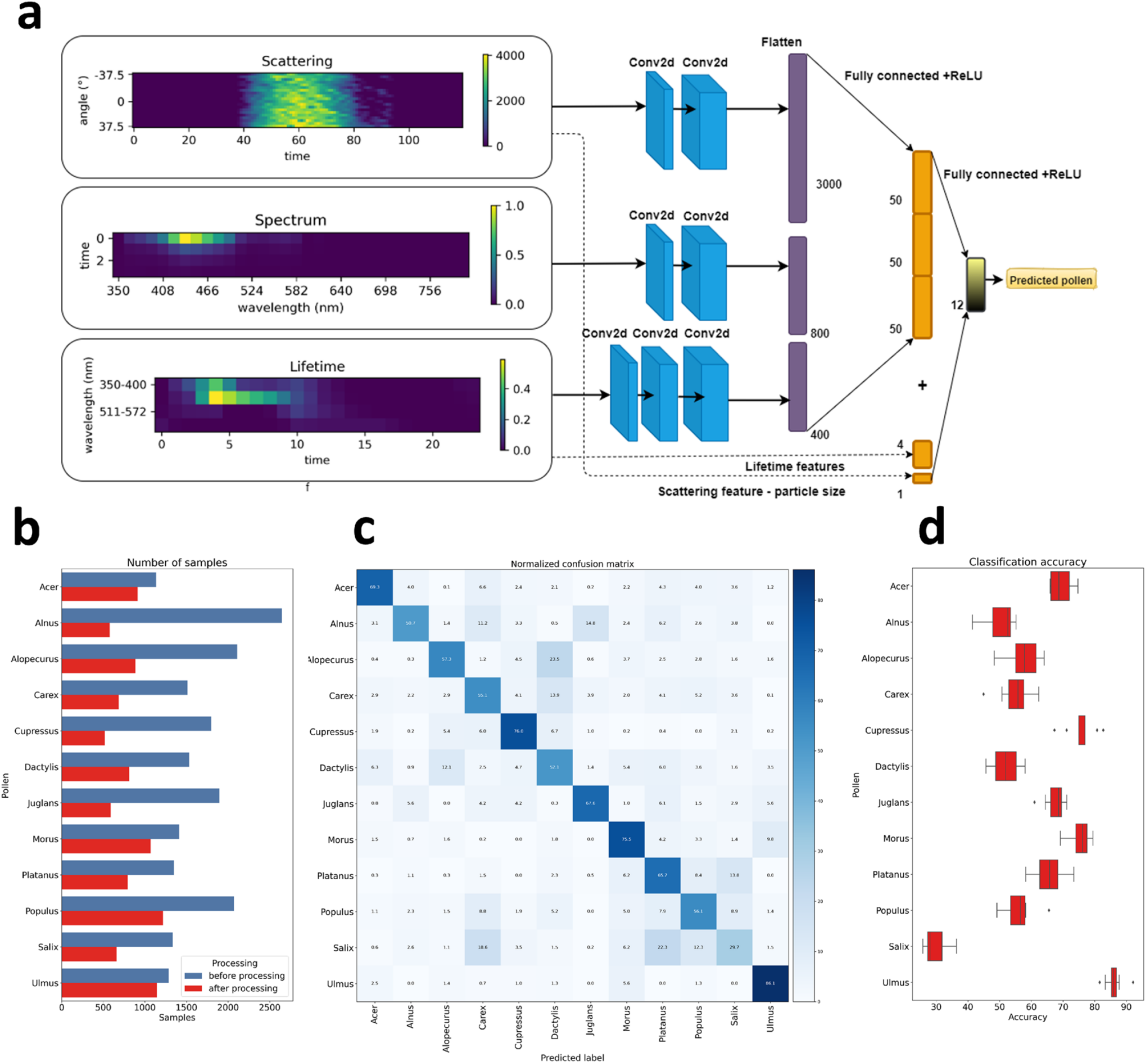
(**a**) Input sample and CNN architecture. (**b**) Number of samples before and after filtering. (**c**) Averaged confusion matrix across 10 experimental runs. (**d**) Variability in accuracy across pollen classes in 10 experimental runs.

CNNs have been proven to be able to classify many pollen types with an accuracy that varies depending on the number of classes to be distinguished, the number of samples available for training, data preprocessing, etc. ^8,11,31^. Our multimodal CNN ^11^ (Fig. 2a) takes at the input preprocessed data coming from three Rapid-E measurements modalities (spectrum, lifetime and scattering), extracts features with convolutional layers for each modality separately and equalizes the sizes of features thus preventing bias in model decision based on the number of features per modality. Features are then concatenated, along with five more features, one representing estimated particle size and the other representing the ratio of lifetime intensities measured at the four spectral ranges to their maximum. Finally, concatenated features are further processed with one fully-connected layer and classified with the log-softmax activation function.

Before training data preprocessing is necessary to remove the noisy samples and align the measured signals. Figure 2b provides information on number of samples before and after preprocessing steps. We can observe that some pollen classes have much more discarded samples than others that can be consequence of the calibration process.

Model training, validation and testing pipeline was performed 10 times for an assessment of model accuracy and its variation. Obtained averaged accuracy in discriminating the 12 examined classes is 63% with variations only in decimals. Normalized and averaged confusion matrix (Fig. 2c) further uncovers which pollen classes are better separated than others and where the errors occur. The best classification is achieved for the following classes Ulmus, Cupressus and Morus, reaching the model accuracy of 86.1%, 76% and 75.5% respectively. Model poorly classified Salix pollen particles with the accuracy of 29.7% by confusing them with particles of Platanus, Carex and Populus pollen. If we examine the standard deviation of model accuracy across the classes we can observe that for some pollen classes (e.g. Alopecurus, Dactylus) it went up to 5% (Fig. 2d). Since the overall accuracy does not change in different training-test splits we can conclude that improvements in some classes led to decline of the accuracy of others implying that maximal results have been achieved with the model trained on current data.

### Instance level explanations

Once the model is learned a question emerges - how the classier makes decisions for particular instance. Term attributions is common in model interpretability and multiple attribution algorithms are associated with it. Algorithms can rely on different principles to quantify attributions such as gradients ^23,46,47^ or perturbations ^48–50^. For our study we selected integrated gradient attribution method ^23^ that uses the input’s gradients after back-propagation and does not require modification of the original network. As our network is multi-modal we adjusted xAI implementation ^51^ to enable multiple inputs into the network.

**Figure 3:**
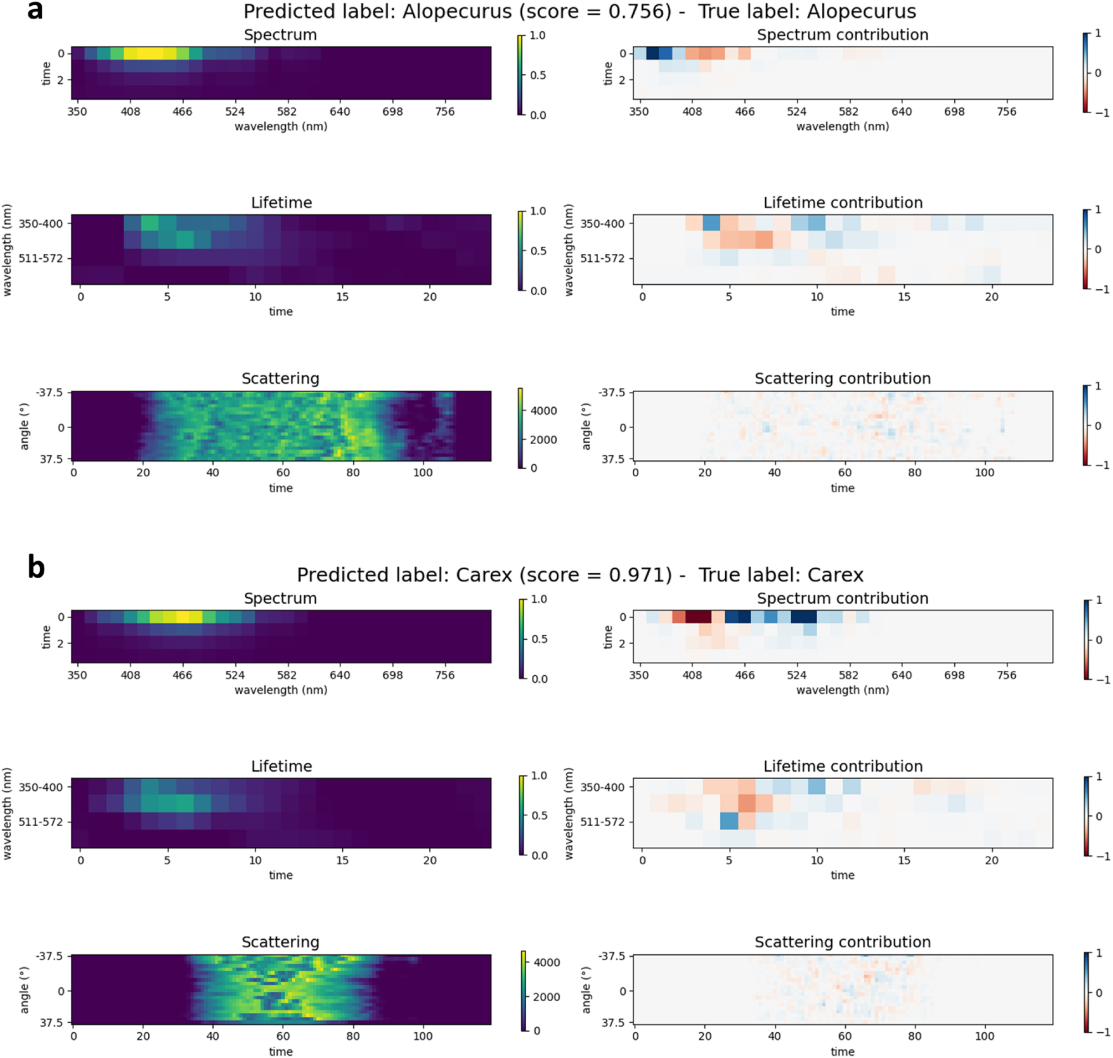
Instance level explanation for two pollen samples of class (**a**) Alopecurus and (**b**) Carex. Input data (graphs on the right) are coupled with derived explanations (graphs on the left) for easy inspection of which part of input contributes the most to the final decision with colors denoting positive or negative contribution.

To illustrate how our model made decision for particular pollen samples Fig. 3 presents two examples of instance level explanation. The images on the left in Fig. 3 represent input samples from different data modalities, while the images on the right are corresponding attributions derived by integrated gradient approach. Intensity of the features in the attribution images corresponds to the impact that particular feature has on the classifier decision, while color denotes whether the impact is positive or negative, i.e. blue or red. The first example (Fig. 3a) refers to the correct classification of Alopecurus pollen grain with log probability score of 0.756. We can observe that strong positive attribution is related to spectrum data in range of 350 to 393 nm, measured at t = 0, with spectral response at 367 nm being the most important from the decision aspect. Observed spectral responses in wavelengths above 408 nm reduced the confidence that pollen sample belongs to Alopecurus class, while other parts of spectrum data modality were irrelevant for the decision. Attributions from lifetime data modality imply that model used ranges of 350–400 nm and 420–460 nm to decide pollen type for particular sample, while other two ranges were less relevant. From the aspect of time in lifetime signals, important features are spread across indexes 3 to 12. Since lifetime data undergo prepossessing that includes alignments of the maximum values we have in all samples maximum at time index 4, and starting from index 5 we have lifetime decay. High positive contribution comes from measured response at 350–400 nm prior to reaching maximum. Importance of scattering features is spread over entire range where signal is detected, having many positive and negative attributions that cumulatively impact the final result along with other modalities. The second example (Fig. 3b) is related to pollen grain of Carex that was correctly predicted with log probability score of 0.971. Spectrum attributions unveiled strong positive impact of measured signal at 451, 466, 524 and 538 nm, while measurements in range of 393 to 422 nm had opposite effect. Nevertheless, final prediction when attributions from all data modalities are joined is made with high confidence. Interestingly, measured maximum in spectral range 511–572 nm of lifetime data positively contributes to the decision that pollen grain is Carex.

Presented examples provide valuable insights of correct class predictions, but such visual inspection can also shed light on misclassification patterns and noisy samples (See Supplementary Fig. 1). Overall, instance level explanations are useful, however it is hard to detect global patterns just by inspection of attributions instance by instance, particularly for our case where signals are complex interplay of timely resolved spectral and scattering responses. Therefore we need some global overview over explained individual predictions.

### Model level explanations

**Figure 4:**
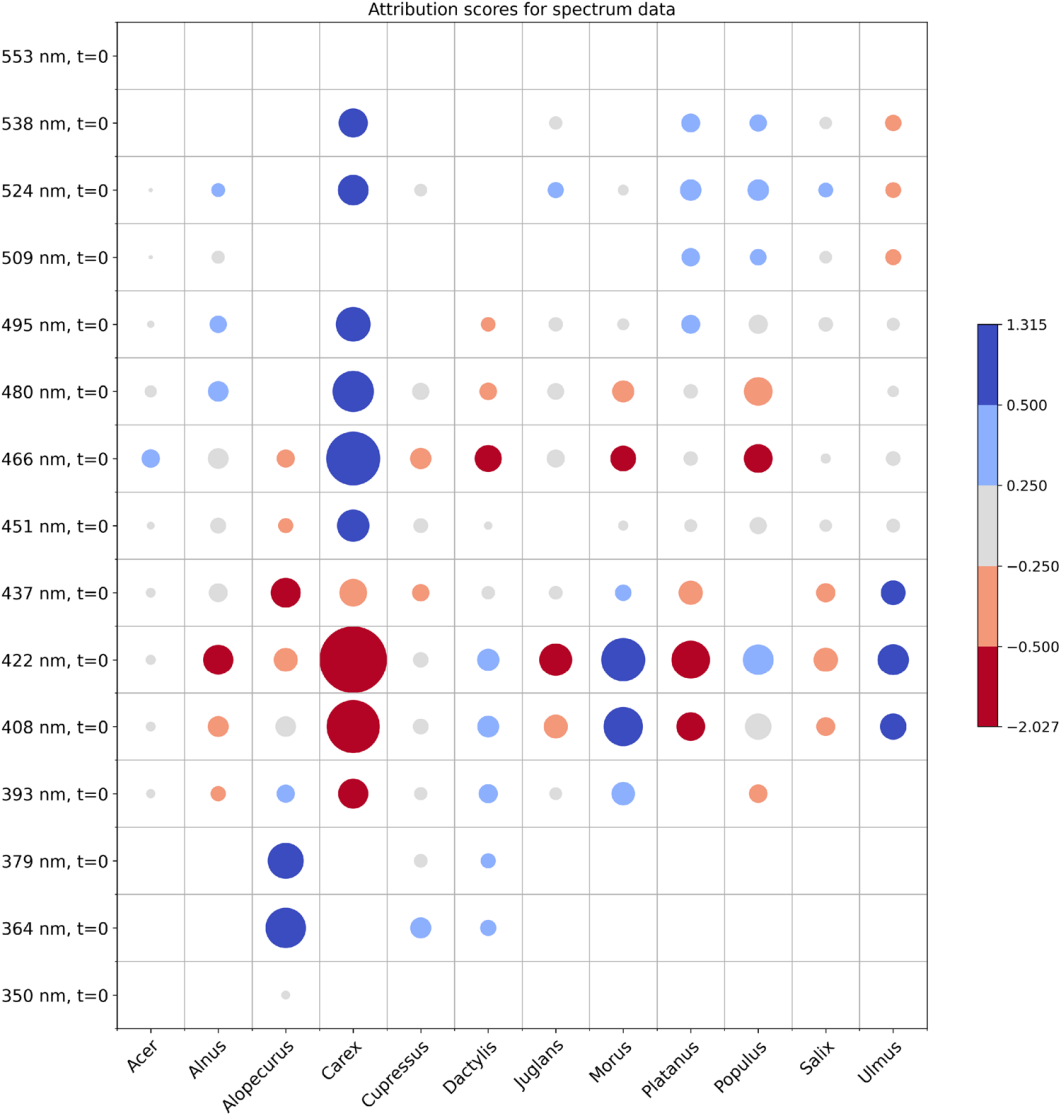
Aggregated attributions of spectrum features. Circle radius correspond to mean absolute attributions of test instances, while color encoding comes from mean value.

Model level explanations aim to uncover how the model behaves on all of data samples. This global explanation provides a holistic view of a model’s behavior. Different aggregations of knowledge from instance level explanations can provide valuable insights and biases of the model ^52^. Here we aggregated all decisions made on test instances for each pollen class aiming to extract high-level patterns. This allowed us to inspect how feature values contribute to the decisions and rank features within each data modality. We focused on top ranked features that drive the decision process. Top 10 ranked features within spectrum data modality (Supplementary Fig. 2) predominately contains spectrum responses measured at t = 0. Only two pollen classes have in top 10 attributions measurements at t = 1, in particular Alopecurus at 379 nm and Morus at 422 nm. Both have the same pattern in t = 0, but stronger. To get more condensed view over important features we grouped information into symbolic heatmap with circles representing averaged attributions of test instances per pollen class (Fig. 4). Circle radius is proportional to the mean absolute attribution of particular feature in predicted pollen class. Colours encode the information on mean attribution, preserving thus the sign of the impact. We distinguish five levels: strong positive, positive, mixed, negative and strong negative, denoted with blues, gray and reds.

**Figure 5:**
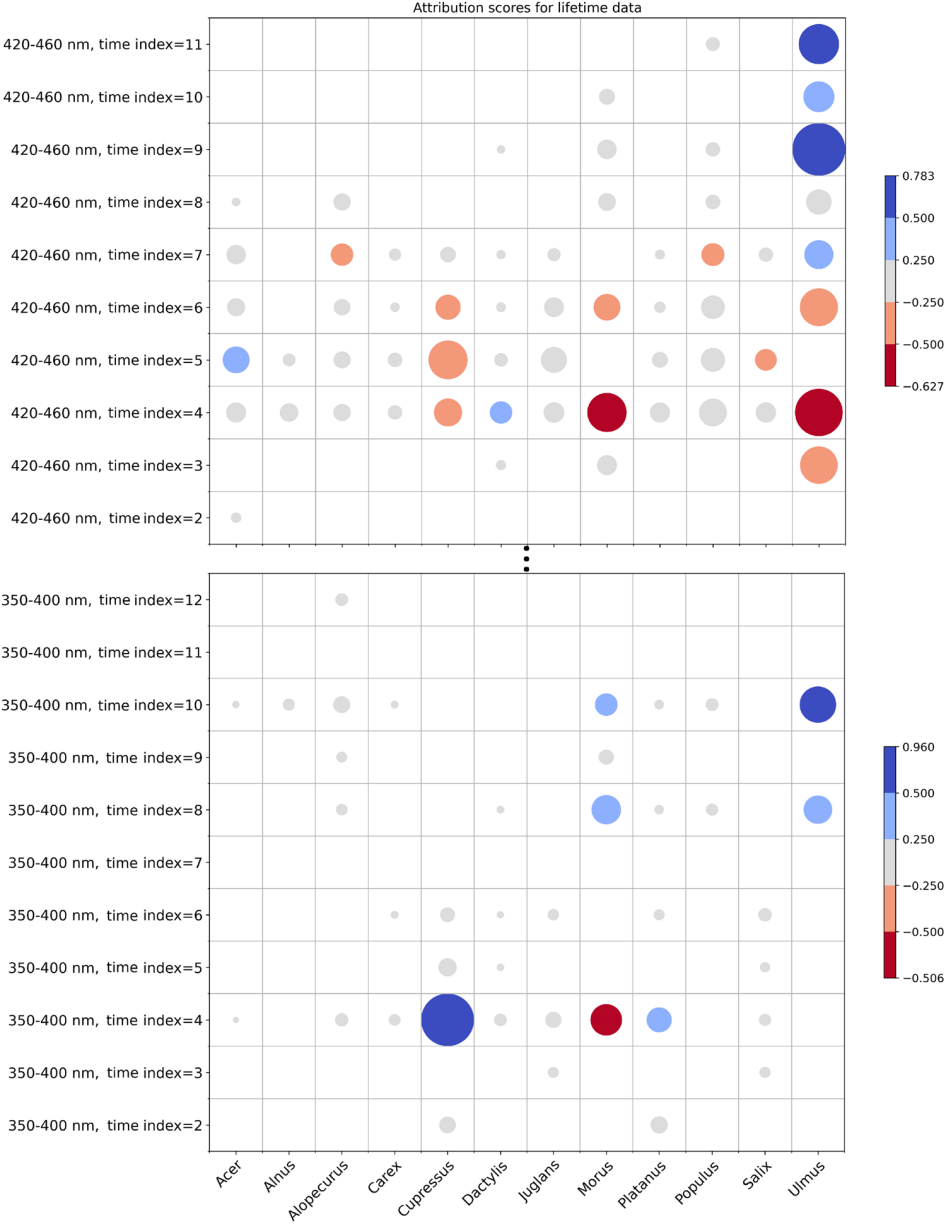
Aggregated attributions of lifetime features. Circle radius correspond to mean absolute attributions of test instances, while color coding comes from mean value.

Top 10 ranked features within lifetime data modality (Supplementary Fig. 3) uncover that spectral range 420–460 nm is the most frequent in highly ranked features, followed by 350–400 nm and 511 and 572 nm ranges. Interestingly, features corresponding to 672–800 nm appear in top 10 ranked features only for Alnus. We can further observe that the most important measurements are those around lifetime maximum, i.e., time index 3–7, where 5 corresponds to lifetime maximum. Symbolic heatmap with circles (Fig. 5) further aggregates information of class level summary plots for lifetime data modality.

Scattering data modality is harder to explain due to higher number of features and noise in light refraction due to particles positions variability during passage through a laser beam and particles irregular surface and shape. However there are evidences of relationships between laser light scattering and physical properties of airborne pollen ^53^, such as positive relationship between grain size and the intensity of forward scattering and surface roughness and the light scattering ratio between side and forward scattering. Scattering data of Rapid-E device mainly records side scatter that captures information on pollen surface structure. Looking from the aspect of our features, angle of $-37.5^\circ$ is more towards forward scattering (somewhere between side and forward), $0^\circ$ is side scattering and $37.5^\circ$ is more towards backward scattering (somewhere between side and back).

**Figure 6:**
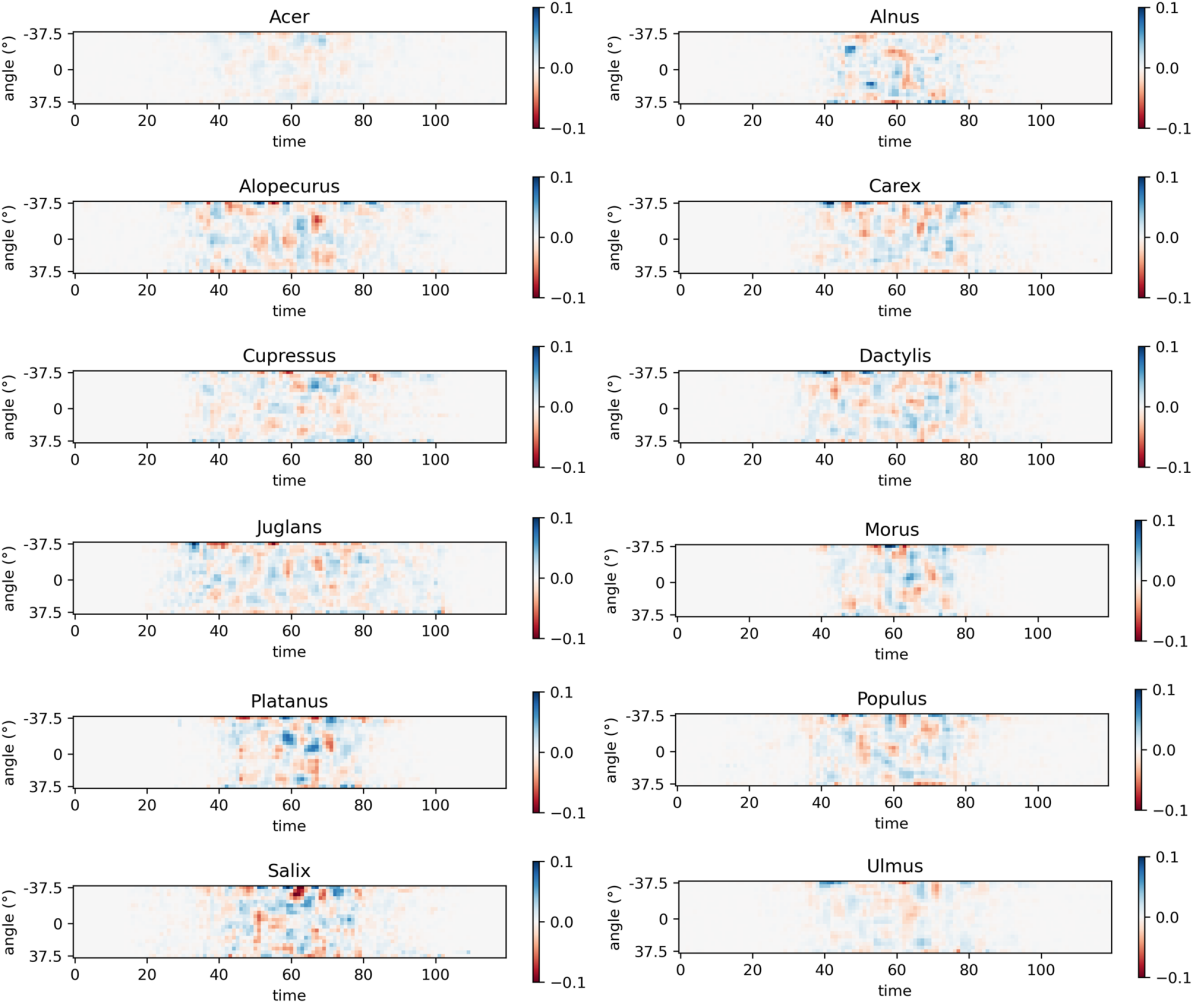
Average contribution in predicting pollen classes across scattering data. Blue and red colors denote positive or negative contribution.

Based on obtained attributions for scattering data, we discovered that values are spread more across the features. Still, top 10 ranked features (Supplementary Fig. 4) mainly correspond to the angle $-37.5^\circ$ that refers to recorded scattered light on the entrance of the particle to the detector and as previously explained could by partially related to the particle size. With scattering data attributions are further spread to the other features and we could not extract strong patterns as those from spectrum and lifetime data were high attributions are concentrated in top ranking features. Therefore we visualized mean attributions to inspect all features together for each pollen class (Fig. 6). Blue color denotes parts of the scattering that contribute positively in predicting observed class, while red negatively. Notable pattern emerges for Juglans pollen class. Compared to the other classes its attributions appear in wider time window. Since detected signal duration is affected by the particle size and shape ^9^ we note that network uses this pattern in classifying Juglans grains. From the attributions values we can observe that model relies less on scatter data in predicting Acer and Ulmus, while for other classes patterns related to the pollen surface influence final decision on pollen class.

Ranked lifetime derived features (Supplementary Fig. 5) based on their attributions showed that feature denoted as lt feature 3 (calculated from lifetime signal in 511–572 nm spectral range) is the most important from this set of features for the majority of pollen classes, followed by lt feature 1 and 2.

Finally, attributions of scattering feature characterizing the particle size (Supplementary Fig. 6) demonstrated that network utilized this information for decision making in case of bigger pollen classes of Juglans, Alopecurus, Dactylis and Carex, although for the last less than expected, since Carex is the biggest pollen in examined group (see Fig. 1 in Data Sources and Supplementary Table 1 with measurements), but we discovered that the actual estimate of the size from measured scattering signal was smaller.

### Knowledge extraction from fluorescence spectroscopy measurements

**Figure 7:**
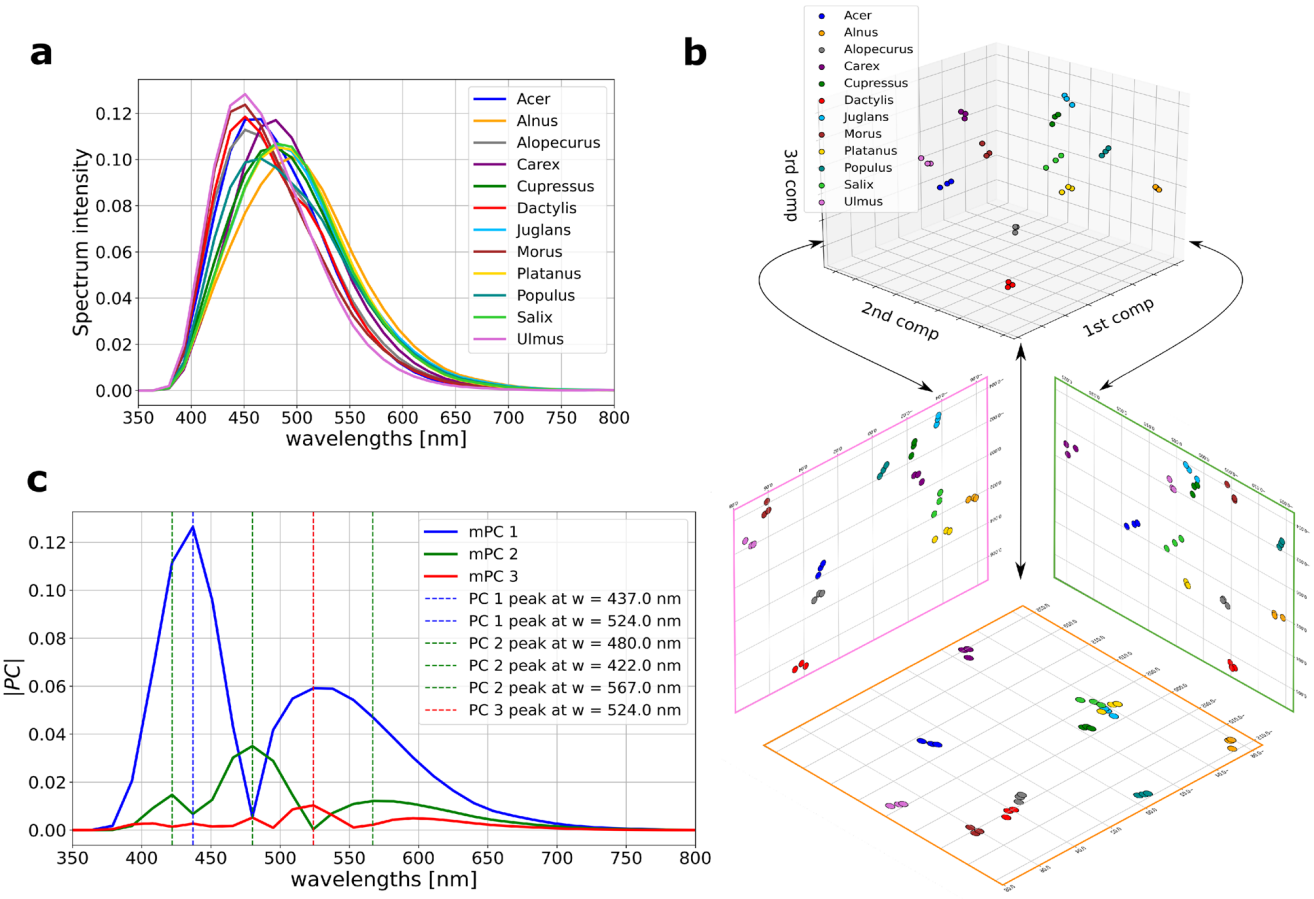
Fluorescence spectroscopy measurements. (**a**) Averages of transformed reference spectrum for each pollen variety. (**b**) Representation of spectra from (**a**) in 3D and 2D latent spaces formed by PCA. (**c**) Magnitudes of PC coefficients with particularly specified prominent peaks and their corresponding wavelengths.

The transformed reference spectral measurements (Fig. 7a) for each pollen variety, aligned with the domain wavelengths of Rapid-E spectral measurements, are analyzed using Principal Component Analysis (PCA) ^54^. The 99$\%$ of variance present in the transformed reference spectral signals is explained with three principal components (PC).

From the depicted 3D and 2D representation of the latent space and projected transformed spectral measurements (Fig. 7b), a clear separability is obvious among two groups of pollen varieties which are resulted from the difference in spectrum mean of the transformed spectrum of pollen. The first group contains Ulmus, Morus, Dactilus, Acer, and Alopecurus, while the second group is formed from the following pollen varieties: Salix, Platanus, Cupressus, Juglans, Carex, Populus, and Alnus. Although in 3D representation seems that projected transformed reference spectral measurements for all pollen varieties are non-overlapping among themselves, in 2D representation this is not the case. Even for three measurements per pollen variety, the overlapping is present in 2D latent space among the projected spectrum of Juglans, Platanus, and Salix in the plane formed from the first and the second PC, and among Cupressus and Juglans in the plane formed from the second and the third PC. Additionally, significant proximity in 2D latent space is observed among Dactylus and Alopecurus.

From the magnitude of PC coefficients, a contribution of spectral measurements on latent space creation, at each of the 32 wavelengths defined by the Rapid-E device was obtained. In Fig. 7c magnitude of PC coefficients are plotted with annotation of wavelengths at important peaks for each PC.

## Discussion

When using machine learning models, possibility to validate the decisions of a model with domain knowledge is always beneficial, but to accomplish that we need xAI for unveiling the decision making of the learned model. Domain knowledge relevant for airborne pollen classification model is interplay of spectroscopy, the physics of scattering and pollen biodiversity. With additional reference data we tackled the challenge of explaining deep learning model for pollen classification.

Measured reference spectrum data which is processed to match coarser Rapid-E spectral resolution was further projected to the space of three the most important principal components to allow visual inspection. Proximity of different classes in that space implies that Rapid-E could hardly resolve these classes only by spectrum modality due to additional noise coming from Rapid-E data single airborne particle acquisition process compared to the laboratory bulk pollen measurements. The high proximity and overlapping between the projected spectrum of Platanus and Salix is also visible in the xAI derived spectrum attributions in the fact that only significant feature having positive attribution for Salix (spectrum at 524 nm, t = 0) overlaps with Platanus. Scattering attributions of those pollen classes differ to some extent, but eventually not enough as reflected on the obtained confusion matrix in Fig. 2c. Also, proximity among Dactylus and Alopecurus, which have the similar shapes and mean values of the transformed reference spectrum as well produce higher rates of the misclassification. On the other hand reference spectrum of Juglans and Salix are likewise highly similar, what aligns with obtained xAI spectrum attributions, but here scattering modality and derived size feature help in separating these classes. Furthermore, PCA of the reference spectral measurements uncovered combination of features that form each principal component. From the highlighted wavelengths pointing on local maximums, three of them at 422 nm, 437 nm and 480 nm are also listed in Fig. 4 with significant contribution on the model classification. Highly ranked lifetime features from range 420–460 nm align with information coming from reference data analysis, where this range stands out as a part of PC1. On the other hand, network discoverers more patterns to separate pollen classes in the range 350–400 nm, than in 511–572, while reference measurements imply that more data variability is explained reversely.

For the cross examination of xAI attributions of scattering data modality we had only measured mean sizes for examined pollen classes and knowledge from literature. We discovered that xAI attributions of the scattering derived size feature positively correlate (61%) with reference size measurements which demonstrates that network utilized size feature. Since time resolved scattering captures also information about the surface morphology and shape of aerosol particles ^9^ we were able to identify pollen classes that based their decisions of relevant part of scattering image, but here additional reference data would be beneficial (e.g. pollen surface roughness estimated from scanning electron micrograph images ^53^). Furthermore, to extend our understanding of scattering contribution other xAI techniques should be explored such as calculating class saliency map ^55^ or a coarse localization map highlighting the important regions in the image for predicting the given class ^35^. Understanding the scattering information is highly challenging also in view of the level of hydration that can have effect on pollen morphology. The classification model analyzed in this study is aiming to identify pollen suspended in the atmosphere which is notably dehydrated pollen and therefore shrivelled. Degree of shrivelling, and resulting size change, could affect performance of the model utilizing scattering signal. It is known that pollen dehydration and subsequent rehydration occurs within minutes ^25^ and the character of this process is unknown. Specific hydration level pollen could be more easily classified using scattering signal but to evaluate such hypothesis controlled conditions in the laboratory would be required.

xAI capability to reason behind the model’s predictions and to identify the features that the model considers the most important can be further used for a variety of tasks, such as model debugging, model improvement and decision-making support. Through xAI we seek for the validation by evaluating whether domain knowledge is being captured by the algorithm. If some of the explanations match with domain knowledge there is more trust in model’s decisions and further setting the hypothesis around new knowledge discovered by xAI. In our experiments xAI opens new avenues of the research. Instance level explanations allowed us to inspect individual errors of classification, where we discovered that some samples were not acquired well by instrument, but still passed all standard filtering steps. This knowledge could be used for designing new filtering approaches for reducing the noise. Learned model is a result of underlying optimization and at the global level we can observe how learned model prioritizes different areas in the spectrum, lifetime and scattering data for different classes. Comparison of different models on the same data, or the same models on different data (e.g. increased number of pollen classes) should be examined not only from the aspect of the accuracy, but also from the perspective of understanding changes in the underling decision making process. This is especially important for advanced deep learning models build with more complex architectures ^31,32^ and for groups of pollen types, as chosen in our study, that have significant overlapping in flowering seasons and were temporal weighting classification ^11^ can not help but classifier itself need to improved. Finally, as results pinpoint which parts of the input data are not relevant and which are highly relevant for the classification, xAI could guide sensors design towards new prototypes of the instruments. Potential improvements could be achieved by increasing the resolution of the measurements in the critical ranges for classifying hardly separable pollen classes and reducing the complexity of sensing by removing elements that do not contribute to the decision making.

## Conclusion

Our study results on 12 pollen classes showed that spectrum data modality strongly influences the decision through condensed range of features in a range of 364–538 nm, mainly measured at t = 0. Lifetime complements spectrum attributions allowing a few pollen classes distinctive patterns not available in spectrum, while attributions of scattering data are spread over wide range of features where scattering signal exists. Findings align well with collected reference data and pinpoint how specific pollen classes are successfully separated while others not. In conclusion, we consider that xAI is a valuable support for explaining models for automatic pollen classification. Presented xAI methodology can be further applied on other types of the instruments for automated pollen monitoring system e.g. Swisens Poleno that combines holographic images fluorescence intensity, lifetime, and light scattering ^30^ or BAA500 ^56^ based on image processing of microscopic slides with pollen grains coupled with deep learning model ^57^. Moreover explainable AI can help to derive insights on how to design new instruments optimized to classify targeted types of pollen.

## Methods

### Collection of pollen and measurements

Dry pollen grains from 12 plant species were collected in Petri dishes directly from the flowers during their natural release and left to dry at room temperature. Plant material used in this study is pollen from common anemophilous plants which is naturally produced and released in atmosphere in large quantities. No plants or their parts were damaged during this process and all used methods were in accordance to relevant regulations.

A small quantity of material from each pollen sample has been put on microscopic slide and embedded in Eukitt, a quick-hardening mounting medium that allowed us to confirm sample purity and measure average diameters (based on 10 measurements at x400 magnification) as expected for dry pollen grains. The samples represent the expected variability of airborne pollen regarding the size of the particle, surface characteristics and chemical composition.

In order to have a reference data set, the fluorescence spectroscopy of dry bulk pollen were measured by the Fluorolog 3 Model Fl3 221 Spectrofluorometer System, supplied by HORIBA. The system is equipped with a 450 W high-pressure Xe lamp and a photomultiplier tube. After excitation at 337 nm, fluorescence emission spectra of pollen samples were recorded in the range from 350 to 800 nm with 1 nm spectral resolution using a quartz optical fiber (4 mm effective diameter) at a distance of 2 mm ^58,59^. Both slits were fixed at 3 nm for excitation and emission beams, and the integration time was 0.1 s. Each sample representing bulk pollen of one class was measured 3 times. FluorEssence 3.5 software (Horiba Scientific, Kyoto, Japan) was used to process all of the measurement data.

### Processing of reference fluorescence spectroscopy measurements

Since the Rapid-E device has a coarser spectral resolution (14.51 nm), resulting in 32 spectrum measurements in the range of 350–800 nm, the reference spectrum signals need to be transformed to be comparable with the obtained spectrum signals from the Rapid-E device. Due to the spectral resolution of reference measurements of 1 nm, all the defined 32 wavelengths by the Rapid-E device are found among reference wavelengths. Transformed reference spectrum signals are created by filtering the reference measurements using the rectangular kernel with the half-width equal to the spectral resolution of the Rapid-E device, and then by sampling the filtered signal at defined 32 wavelengths ^60^. Therefore, for each pollen variety we obtained three transformed reference spectrum signals, for which averages are shown in Fig. 7a.

### Rapid-E data preprocessing

The collected dataset is first filtered out to exclude wrong measurements. Only particles with maximum spectrum intensity greater than 2500, scattering image size smaller than 450, maximum lifetime index between 10 and 44, and four maximum spectrum indices between 3 and 10 are included in the analysis. The scattering image is centered around the maximum intensity and then cut to take 60 pixels to the left and right, obtaining an image of 20 $\times$ 120 dimension. At the same time, the multiple recorded fluorescence signals are stacked on top of each other to obtain spectrum image and lifetime image of dimensions 4$\times$32 and 4$\times$24 after preprocessing, respectively. In case of lifetime signals additional alignment of measurements is needed, maximum values are placed at index 4 and signals are than cut to fit size of 24. Specially derived four features from lifetime modality are extracted for each spectrum range and represent sum of signal corrected for the noise in that range normalized by maximum value among four extracted features. Size feature derived from scattering is proportional to the logarithm of sum of pixel values in spectrum image. Data augmentation was not used to increase the training data size since it could affect key properties of recorded signals and thus introduce more noise into data.

### Multi-modal convolutional neural network architecture and training

As illustrated in Fig. 2a tensors representing scattering, spectrum and lifetime data modalities are the inputs along with specially derived features from scattering and lifetime. Scattering image passes through two convolutional layers encompassing batch normalization, padding, 2D convolution followed by dropout, maxpool and ReLU function. One-channel scattering image is transformed into 10 and 20-channel images that are flatten into 3000 features before applying fully connected layers. Similarly spectral data represented as image passes through two convolutional layers with distinction that image is transformed into 50 and 100-channel images producing after flattening 800 features. On the other hand lifetime data are transformed through 3 convolutional layers with kernel sizes 7, 5, and 3 forming 70, 140 and 200-channel images producing overall 400 features. Features from different modalities are further reduced on 50 each through fully connected layer. Finally features are concatenated including also 4 additional lifetime features extracted from initial data and one size related feature from scattering image. This 155 features enter the last fully concatenated layer.

We balanced training set by randomly selecting 500 samples of each class, while the rest of the samples is left to test the model. Out of training set 10% of samples was used for validation. The model was trained using the negative log-likelihood function, and the stochastic gradient descend algorithm with 0.001 learning rate and a momentum of 0.9. Batches for training were balanced, representing mixture of 20 randomly picked samples of each classes. Training was performed in up to 800 epochs depending on the calculated training and validation loss. Experiment run on computer with Intel(R) Core(TM) i5-7300HQ CPU @ 2.50GHz (4 CPUs),  2.5GHz and Nvidia GeForce GTX 1050 graphics card, with 4GB of dedicated memory. It needs  40 minutes to train the network. Overall training-test procedure was run 10 times to obtain mean accuracy and evaluate also stability of the results.

### Explainable framework

The core component of the explainable framework is based on Integrative Gradients (IG) method that is attributing the prediction of a deep network to its input features. IG satisfies the completeness axiom as the sum of the attributions is the difference between the input signal and the baseline. In our experiments baseline is zero denoting absence of signal at detector. IG of *i*th feature can be calculated as:

1 $$\begin{aligned} IG_{i}(x, x^{'}) = (x_{i} - x_{i}^{'}) \times \int _{\alpha =0}^1 \frac{\partial f(x^{'} + \alpha \times (x - x^{'}))}{\partial x_{i}^{'} }d\alpha \end{aligned}$$

where *f*(*x*) represents deep neural network function $R^n \rightarrow [0, 1]$ for input $x =(x_1,\ldots , x_n) \in R^n$.

Calculation can be further simplified with sum of the gradients at points occurring at sufficiently small intervals *m* in *M* steps along the straight line from the baseline to input:

2 $$\begin{aligned} IG_{i}(x, x^{'}) \approx (x_{i} - x_{i}^{'}) \times \frac{1}{M} \sum _{m =1}^M \frac{\partial f(x^{'} + \frac{m}{M} \times (x - x^{'}))}{\partial x_{i}^{'} } \end{aligned}$$

Developed explainable framework uses Captum, a unified and generic model interpretability library for PyTorch ^51^. Our multi-modal convolutional network is examined through this framework. Its forward function takes five tensors as input (spectrum, lifetime and scattering modalities and derived lifetime and size related features).

## Supplementary Information

Supplementary Information.

## Data Availability

The datasets generated from Rapid-E instrument (raw and processed) and analysed during the current study are available in the Zenodo repository, https://doi.org/10.5281/zenodo.7055577. The raw measurements from HORIBA Fluororlog-3 spectrofluorometer are available from the corresponding author on reasonable request.
