# Supplementary material for: Explainable AI for unveiling deep learning pollen classification model based on fusion of scattered light patterns and fluorescence spectroscopy
Source: Sci Rep. 2023 Feb 24;13:3205. doi: 10.1038/s41598-023-30064-6 (PMC9958198; doi:10.1038/s41598-023-30064-6)
Supplement: Supplementary file 1 — Supplementary Information. [file 41598_2023_30064_MOESM1_ESM.pdf]

Explainable AI for unveiling deep learning pollen classification models based on fusion of scattered light patterns and fluorescence spectroscopy - Supplementary Materials

Sanja Brdar[1], Marko Panić[1], Predrag Matavulj[1], Mira Stanković[2], Dragana Bartolić[2], and Branko Šikoparija[1]

[1]BioSense Institute – Research Institute for Information Technologies in Biosystems, University of Novi Sad, Novi Sad, Serbia
[2]Institute for Multidisciplinary Research, University of Belgrade, Belgrade, Serbia

Pollen sizes

Label	Pollen species	Polar view average size (standard deviation)	Equatorial view average size (standard deviation)
Acer	Acer negundo L.	32 (8) micron	22 (4) micron
Alnus	Alnus glutinosa (L.) Gaertn.	27 (1) micron	24 (2) micron
Alopecurus	Alopecurus pratensis L.	36 (2) micron	30 (2) micron
Carex	Carex sp.	52 (2) micron	44 (4) micron
Cupressus	Cupressus arizonica Greene	22 (1) micron	21 (7) micron
Dactylis	Dactylis glomerata L.	34 (3) micron	32 (4) micron
Juglans	Juglans regia L.	46 (3) micron	41 (4) micron
Morus	Morus sp.	22 (2) micron	16 (2) micron
Platanus	Platanus x acerifolia (Aiton) Willd.	22 (3) micron	18 (2) micron
Populus 1	Populus nigra var. pyramidalis Spach	25 (2) micron	22 (2) micron
Populus 2	Populus alba L.	30 (2) micron	25 (3) micron
Salix	Salix sp.	25 (4) micron	17 (1) micron
Ulmus	Ulmus sp.	30 (2) micron	26 (3) micron

Supplementary Table 1: Measured averaged pollen sizes of examined pollen classes.

Instance level explanations - examples of misclassifications

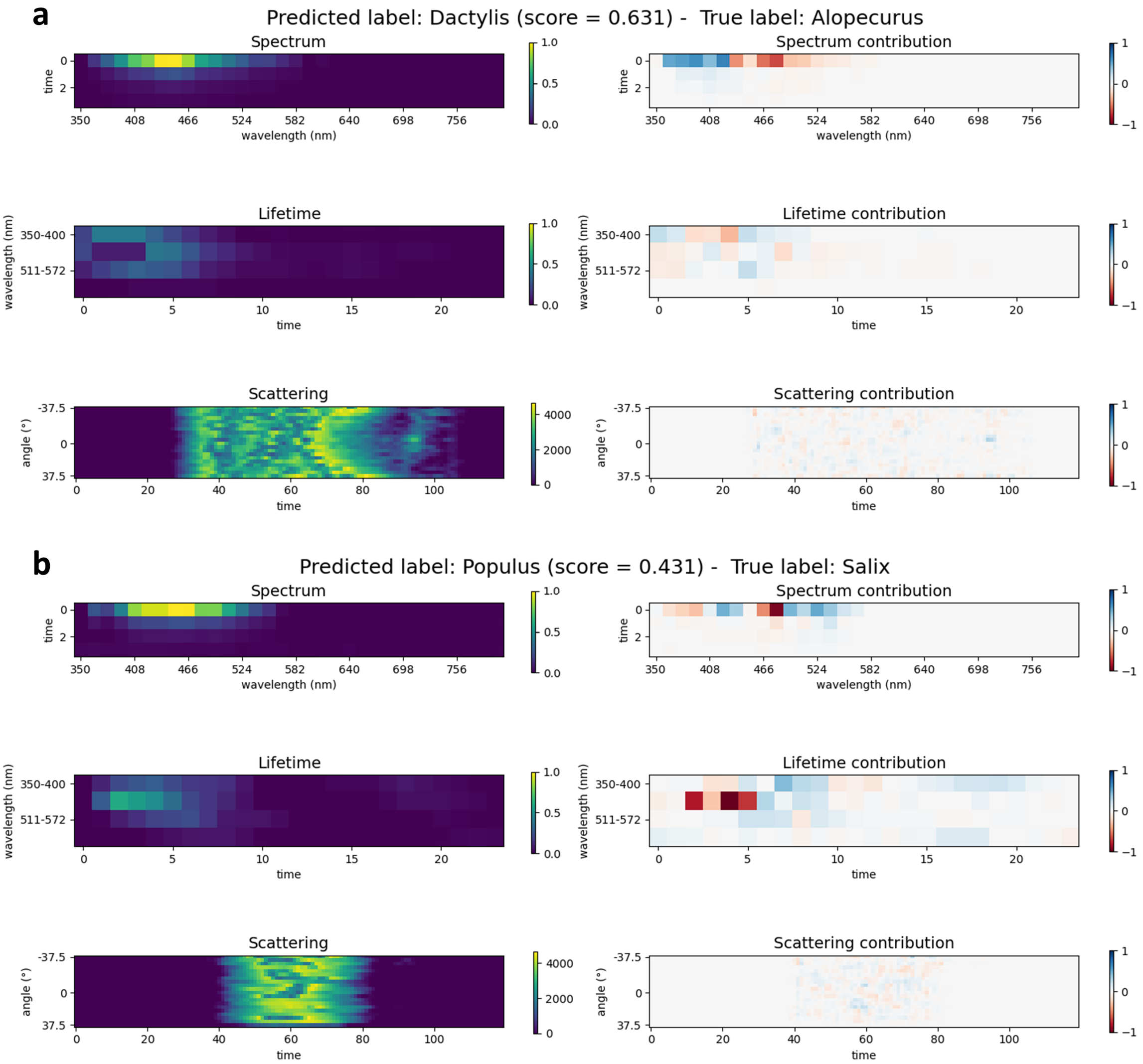

Supplementary Figure 1: Examples where classifier wrongly predicts test instances.

Model level explanation - spectrum attributions

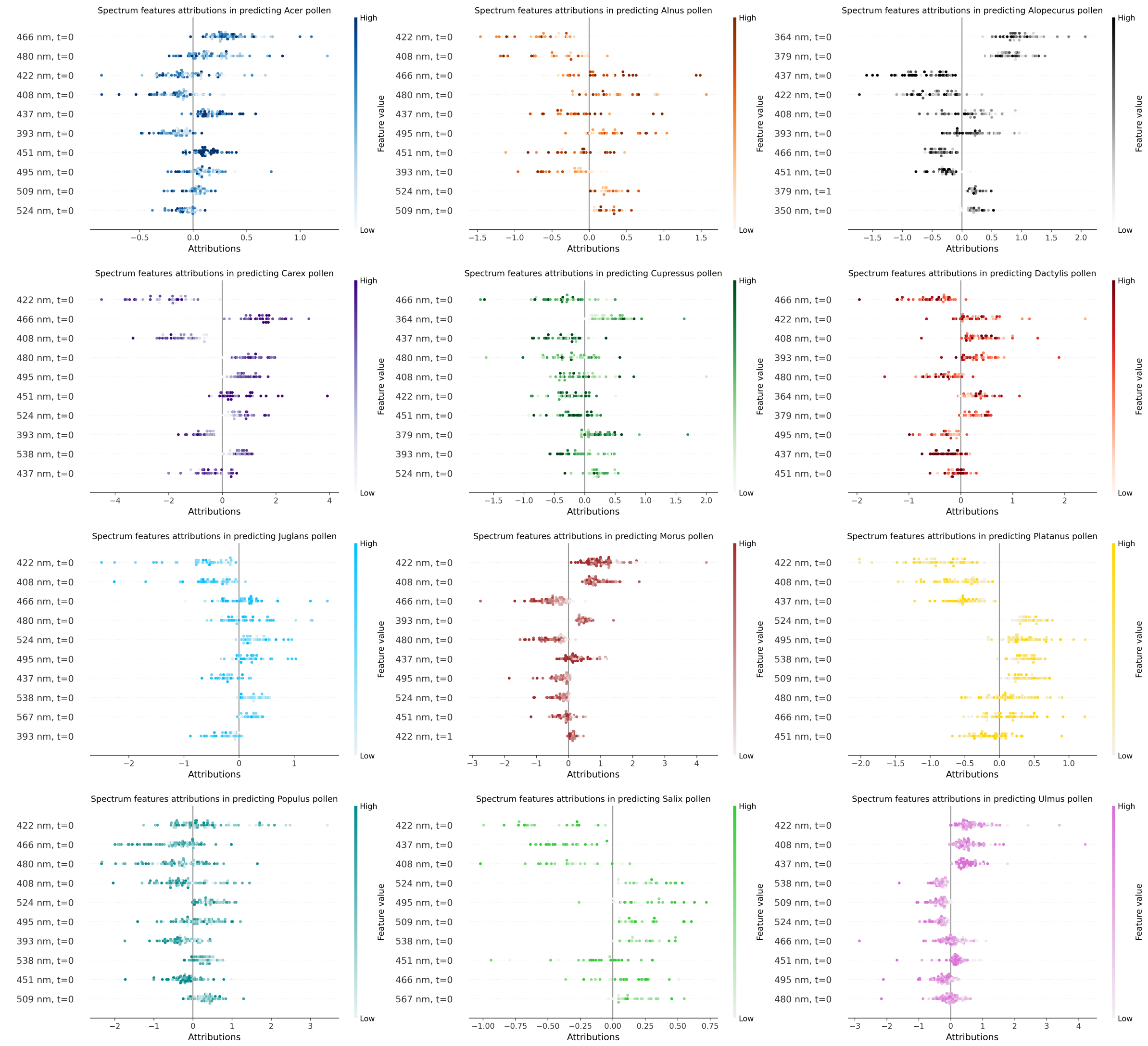

Supplementary Figure 2: Ranked attributions of top 10 spectrum features.

Model level explanation - lifetime attributions

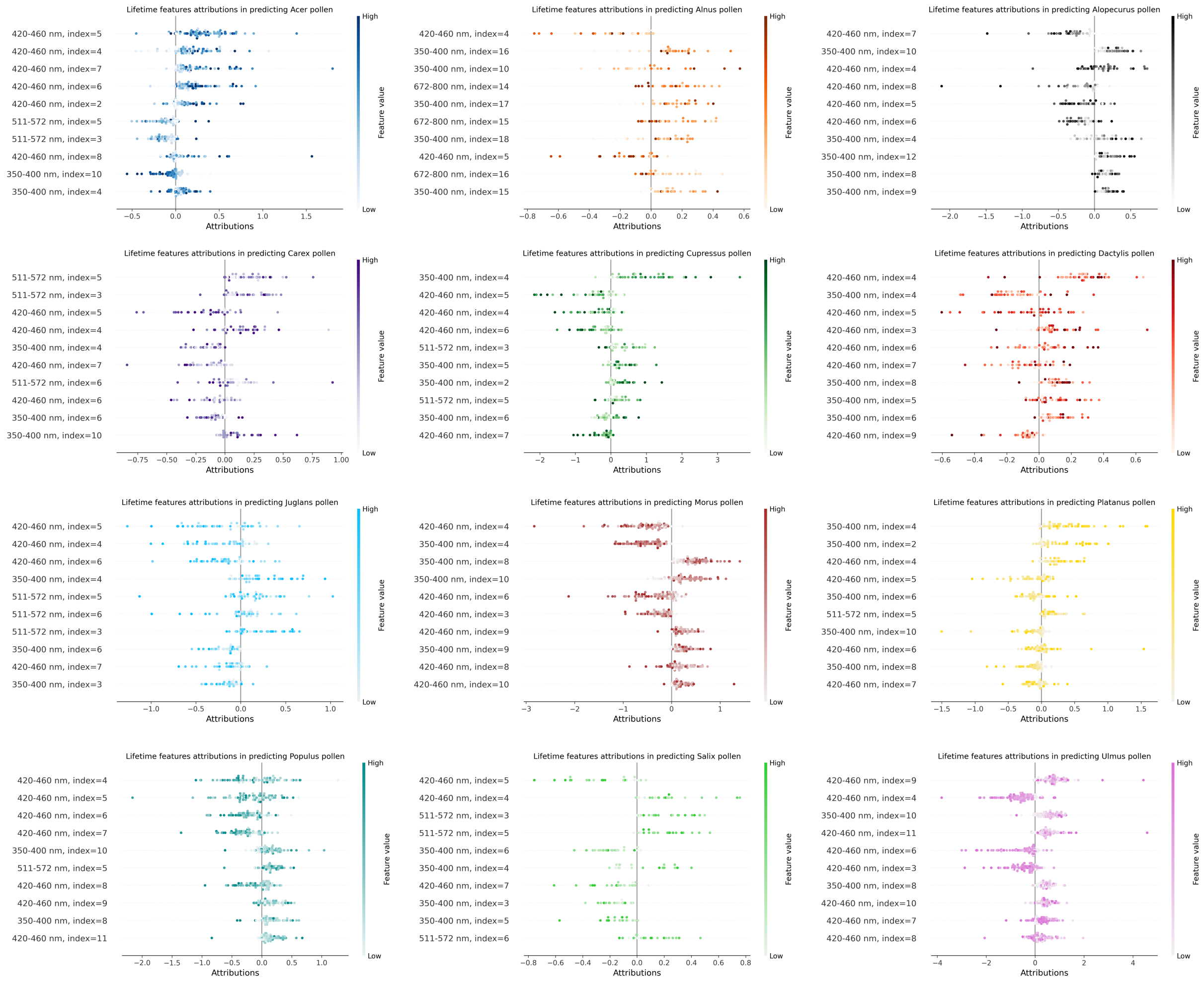

Supplementary Figure 3: Ranked attributions of top 10 lifetime features.

Model level explanation - scattering attributions

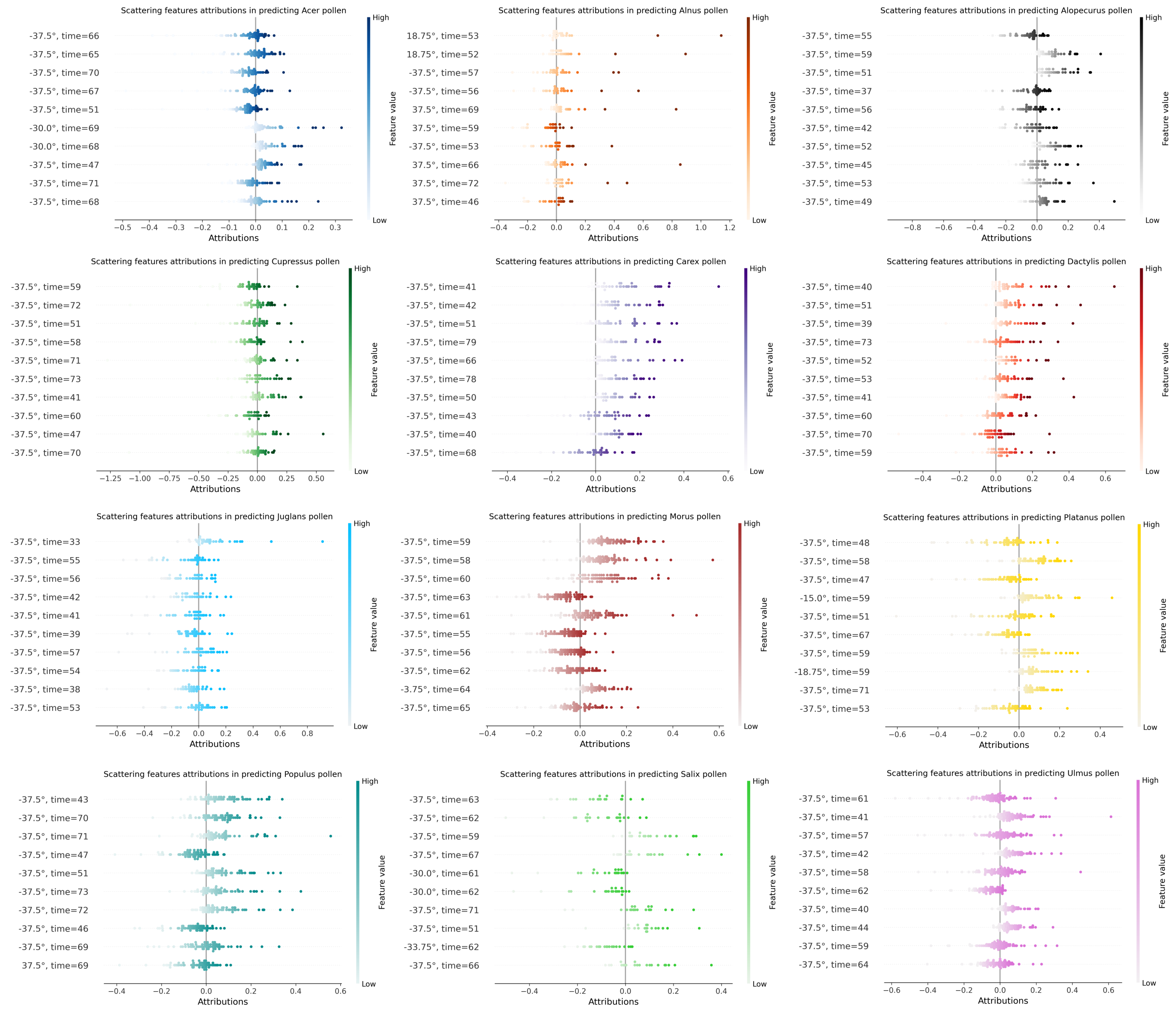

Supplementary Figure 4: Ranked attributions of top 10 scattering features.

Model level explanation - lifetime derived features attributions

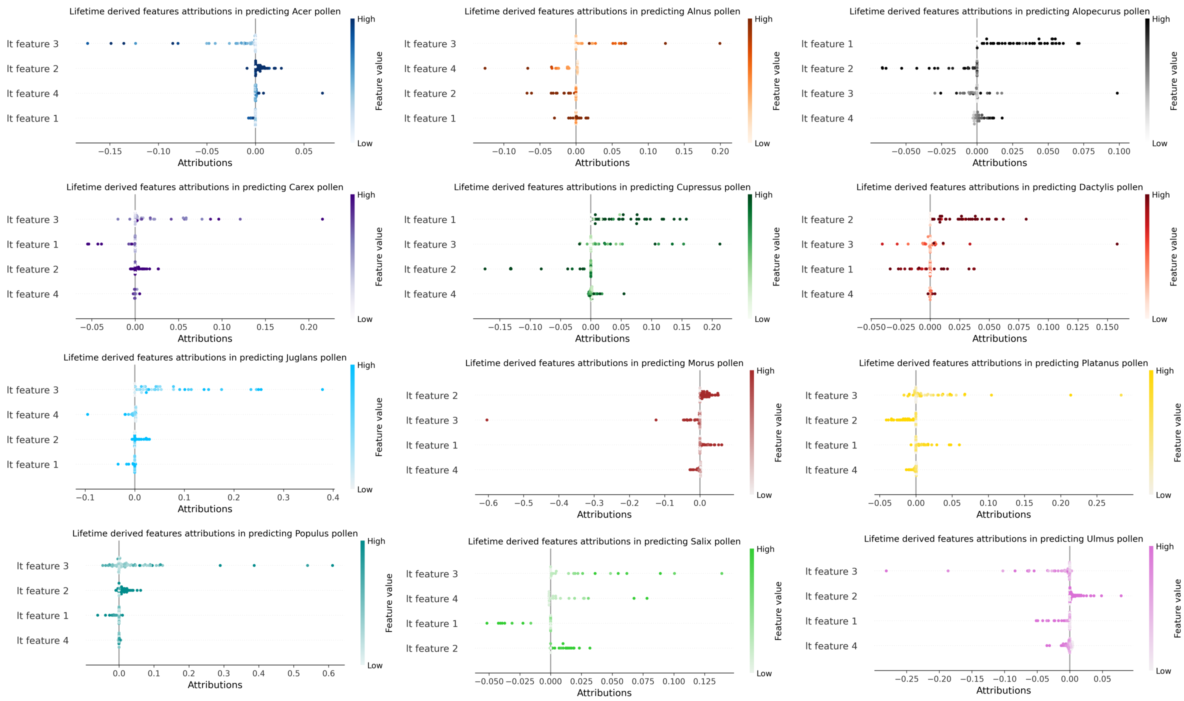

Supplementary Figure 5: Ranked attributions of lifetime derived features.

Model level explanation - size feature attributions

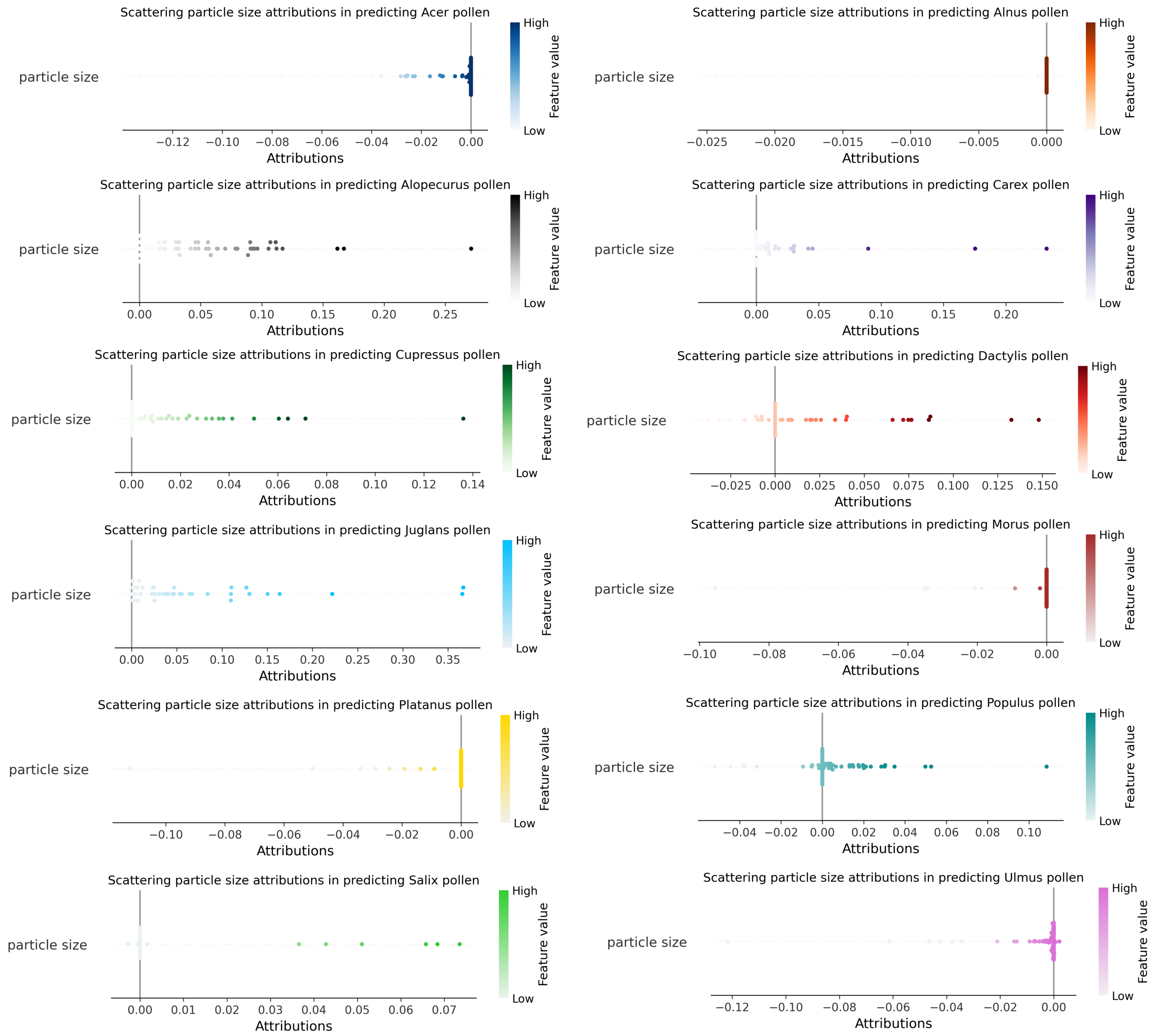

Supplementary Figure 6: Attributions of scattering derived feature - size.
